# Interleukin 10 and Transforming Growth Factor Beta Polymorphisms as Risk Factors for Kawasaki Disease: A Case-Control Study and Meta-Analysis

**Published:** 2019

**Authors:** Farzaneh Rahmani, Vahid Ziaee, Raheleh Assari, Maryam Sadr, Arezou Rezaei, Zeinab Sadr, Seyed Reza Raeeskarami, Mohammad Hassan Moradinejad, Yahya Aghighi, Nima Rezaei

**Affiliations:** 1. Research Center for Immunodeficiencies, Children’s Medical Center, Tehran University of Medical Sciences, Tehran, Iran; 2. NeuroImaging Network (NIN), Universal Scientific Education and Research Network (USERN), Tehran, Iran; 3. Pediatric Rheumatology Research Group, Rheumatology Research Center, Tehran University of Medical Sciences, Tehran, Iran; 4. Department of Pediatrics, Pediatrics Center of Excellence, Children's Medical Center, Tehran University of Medical Sciences, Tehran, Iran; 5. Molecular Immunology Research Center, School of Medicine, Tehran University of Medical Sciences, Tehran, Iran; 6. Department of Pediatrics, Vali-e-Asr Hospital, Tehran University of Medical Sciences, Tehran, Iran; 7. Department of Pediatrics, Imam Khomeini Hospital, Tehran University of Medical Sciences, Tehran, Iran; 8. Department of Immunology, School of Medicine, Tehran University of Medical Sciences, Tehran, Iran; 9. Network of Immunity in Infection, Malignancy and Autoimmunity (NIIMA), Universal Scientific Education and Research Network (USERN), Boston, MA, USA

**Keywords:** Cytokine, Interleukin-10, Kawasaki disease, Single nucleotide polymorphisms, Transforming growth factor-beta

## Abstract

**Background:**

Alteration in serum expression of Transforming Growth Factor-beta (TGF-β) and IL-10 have been suggested to play a role in the pathogenesis of Kawasaki Disease (KD). Inconsistent reports exist on the association of IL-10 polymorphisms with KD susceptibility and Coronary Artery Aneurysms (CAA).

**Methods:**

A number of 110 paediatric patients with KD and 140 healthy individuals were recruited to investigate the frequency of Single Nucleotide Polymorphisms (SNPs) of TGF-β C/T at codon 10 (rs1982073), C/G at codon 25 (rs1800471) and IL-10 A/G at −1082 (rs1800896), C/T at −819 (rs1800871) and A/C at −592 (rs1800872) and their respective genotype and haplotypes. A comprehensive search was performed in MEDLINE and SCOPUS using the keywords of interleukin 10, transforming growth factor beta, and Kawasaki disease. Moreover, previous studies investigating the TGF-β and IL-10 polymorphisms in KD were evaluated. Review Manager Version 5.1 Software was used to perform meta-analysis.

**Results:**

There was no significant association between allelic or genotypic variants in the mentioned polymorphisms in TGF-β or IL-10 with KD or CAA. The only significant haplotypic variant was TC variant at codon 10, and 25 of TGF-β polymorphisms were associated with higher risk of KD. Meta-analysis of a total number of 770 patients *vs.* 1471 healthy controls showed no difference in the frequency of any of the IL-10 genetic variants in KD patients, regardless of the presence of CAA.

**Conclusion:**

Polymorphisms of TGF-β or IL-10 are not associated with additional risk for KD in Iranian population. IL-10 polymorphisms at −1082, −819 and −592 positions are not associated with KD, nor do they predict coronary artery aneurysm formation.

## Introduction

Kawasaki Disease (KD) also known as mucocutaneous lymph node syndrome is an acute systemic vasculitis with strong predilection for coronary arteries 1. KD affects children between 6 months to two years of age and is top listed in the causes of acquired heart disease in children, with up to 50% of children developing cardiac complications 2. These start from mild pericarditis, to myocarditis and Coronary Artery Aneurysm (CAA). Early identification of the disease, especially in young patients and aggressive treatment of patients with additional risk of CAA, with Intravenous Immunoglobulin infusion (IVIG), reduce risk for coronary artery aneurysm formation 3 and subsequent myocardial infarction which is a leading cause of mortality in young adults with chronic KD 4.

During the initial acute stage of KD, markers of inflammation were abundantly found in plasma, helper CD4+ T-cells which dominate the peripheral blood 5. During the first two weeks, acute mucocutaneous manifestations predominated along with fever, increase in serum ESR and CRP levels, and release of markers of endothelial injury, IL-1β, TNF-α, and endothelial microparticles into the plasma 6. Also, the regulatory T cell cytokines, TGF-β and IL-10 upregulate in acute KD but not to comparable levels of proinflammatory cytokines 7. A remission in the proinflammatory milieu denotes transition from acute to subacute stage of the disease 8.

Expression levels of FOXP3, which is a crucial transcription factor of Treg and Th17 cells is suppressed in acute febrile phase of KD, and surges to a peak three weeks after IVIG therapy 8. Interestingly, this late Treg expansion is not reflected in Treg cytokines secretion profile as levels of transforming growth factor beta (TGF-β) and IL-10 diminish gradually with initiation of IVIG therapy and IVIG resistance has been attributed to an initial surge of both IL-10 and IL-17 expression 8. Whether this suggests proinflammatory traits for IL-10 and/or TGF-β in acute stage of KD, or reflects alternative pathways by which IVIG is able to reduce acute inflammatory response, remains to be a question. Failure of this Treg expansion in late KD results in IVIG resistance and poor response to therapy 9 and CAA formation. The double-edged effect of TGF-β expression in KD pathology can be justified by findings that report an initial increase in peripheral TGF-β expression to drive maturation of myofibroblasts from smooth muscle cells of vessel walls and precursors in the circulation 10. The myofibroblasts help maintain the proinflammatory state mainly by secretion of chemokines that confirm the diagnosis of acute vasculitis in early KD and by direct production of inflammatory cytokines, IL-6 and IL-17 11. Termination of acute inflammation is achieved by induction of FOXP3 Treg cells expression and TGF-β and IL-10 induction of a profibrotic state in myofibroblasts and polarization into mature fibroblasts which lose their potential to express TGF-β 12. Local activation of TGF-β signalling might then contribute to the aberrant tissue remodelling pathways that underlie formation of coronary artery aneurysms 7.

A large body of literature support association of polymorphisms of TGF-β signalling pathway with predilection to KD and CAA 13–16. Meanwhile, few reports have addressed genetic variants of IL-10 in KD, and even fewer have investigated *TGF-β* gene polymorphism itself. Single nucleotide variants of proinflammatory cytokines 17, IL-4 18, and association with KD in our region were previously investigated. Also, polymorphisms of TGF-β and IL-10 with common variable immunodeficiency, inflammatory bowel disease, chronic idiopathic urticaria and Systemic Lupus Erythematosus (SLE) have been previously investigated 17–24. Herein, allelic, genotypic and haplotypic frequency of polymorphisms of *TGF-β* (codon 10 and codon 25) and *IL-10* genes were addressed. Considering the discrepancy in results of various studies on IL-10 polymorphisms including A/G at −1082, C/T at −819, and A/C at −592, meta-analytic evidence on association of IL-10 allelic and genotypic variants with KD susceptibility was provided.

## Materials and Methods

### Study design

In this study, 110 Iranian patients (60 males and 55 females) were recruited with acute KD, who were randomly selected from referrals to the Children’s Medical Center, the Pediatrics Center of Excellence in Tehran, Iran, and a group of 140 age-sex matched healthy individuals as the control group was selected. It was a single center prospective observational case-control study. Sample size was calculated based on the following formula 25:

$$\frac{r+1}{r}\times \frac{SD^{2}{\left(Z_{\beta }+Z_{\frac{\alpha }{2}}\right)}^{2}}{d^{2}}$$

where, SD is the expected standard deviation of the variable, extracted from previous literature, d is the expected mean difference between case and control group which can be extracted from previous studies, r is case to control ratio, and Z_β_ and Z_α_ are standard normal variates for power and level of significance, respectively. Adopting a statistical power of 80% (Z_β_=0.84) and significance threshold of 95% (Z_α/2_=1.96), SD and mean differences reported by Hsieh *et al* 26 were used for IL-10 polymorphisms, and Shimizu *et al’s* statistics 27 were used to calculate the sample size for TGF-β polymorphisms.

The diagnosis of KD was primarily made according to American Heart Association Criteria 28, based on the clinical grounds and patients echocardiography at least one month after initiation of symptoms, and also the patients’ most recent echocardiography if available. Patients were excluded if they had any concomitant genetic inflammatory condition including other types of systemic vasculitis, systemic lupus or juvenile arthritis, etc. Among KD patients, 70 of them had cardiac involvement and 10 patients (9.1%) were IVIG resistant. The data were compared to 140 healthy individuals 29. Healthy controls had no personal history of vasculitis, or other rheumatologic, or autoinflammatory conditions themselves or among their siblings or parents.

This study was approved by local ethics committee and institutional review board of Tehran University of Medical Sciences in April 2017. Signed informed consent forms were obtained from parents of all enrolled cases to perform sampling and echocardiography when necessary, and for publication of the results as papers.

### Sampling and genotyping

Five *ml* of whole blood was taken from each participant and kept with EDTA (Ethylene-diamine-tetraacetic acid) until investigation (Lymphodex, Kronberg/Taunus Inno-train, Germany). Genomic DNA was extracted from whole blood, using Phenol-Chloroform method 30. Polymerase chain reaction with the Sequence Specific Primers (PCR-SSP) assay was employed (PCR-SSP kit, Heidelberg University, Heidelberg, Germany) for this study, as explained before 31. The polymorphic sites explored in this study included TGF-β polymorphisms C/T at codon 10 (rs1982073) and C/G at codon 25 (rs1800471) and IL-10 promoter polymorphisms A/G at −1082 (rs1800896), C/T at −819 (rs1800 871) and A/C at −592 (rs1800872).

### Statistical analysis

The analyses were all performed using the Epi Info statistical software (version 6.2, World Health Organization, Geneva, Switzerland). Allele, genotype and haplotype frequencies were estimated by direct gene counting. All of the allele frequencies were in line with the Hardy-Weinberg equilibrium. Frequencies were analyzed using chi-square test or Fisher’s exact test and odds-ratios of 95% Confidence Interval (95%CI). All tests were two-sided and the probability of less than 0.05 was considered statistically significant.

### Systematic review and meta-analysis

#### Search strategy, study selection and data extraction:

For the meta-analysis, original articles investigating frequency of *IL-10* genes and *TGF-β* gene polymorphisms in KD patients were included in English language, with no limitation on publication date or status. A comprehensive literature search through PubMed and Scopus was done by using the key words of interleukin 10, transforming growth factor beta, IL-10 OR TGF-β, KD, and mucocutaneous lymph node syndrome. The search was repeated and updated accordingly to the date of submission (August 2018). To identify additional studies, reference lists and contacted corresponding authors of all the publications included in the present systematic review and meta-analysis were scanned.

**Figure 1.**
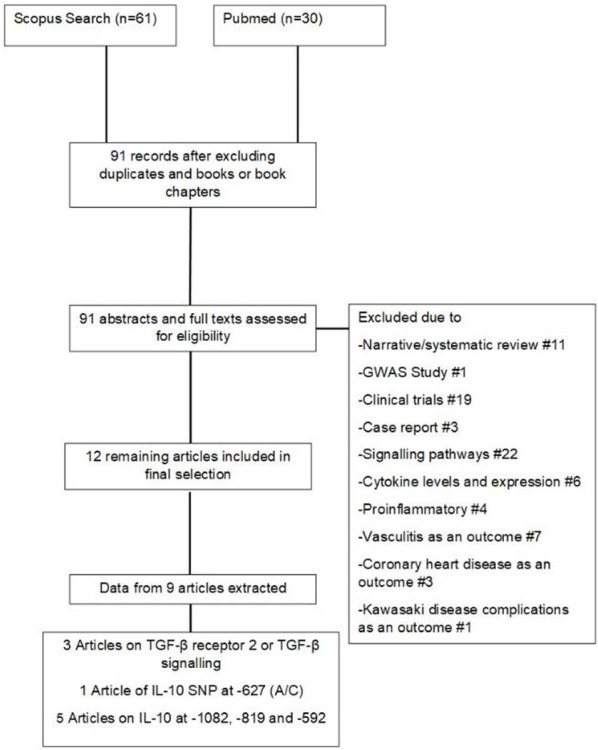
Search strategy and study selection flow chart for single nucleotide polymorphisms of IL-10 at −1082, −819, and −592 and TGF-β at codons 10 and 25.

Two investigators independently reviewed results of 91 studies yielded through literature search according to preliminary inclusion criteria listed above, based on title and abstract. Any disagreement between reviewers was resolved by discussion or referral to a third investigator. Additional inclusion criteria were: 1) diagnosis of KD established by a pediatric rheumatologic disease and/or according to international criteria, 2) availability of either number or odds ratio or relative risk of the alleles, genotypes or haplotypes, 3) primary outcome of the study being KD, CAA or both. Studies on atypical KD, other complications of KD such as heart block or myocardial infarction, and those in which the control group was selected from related or non-related individuals with other cardiac complications, were excluded. Reference stratification and systematic reviews were performed using the SM Software. The following data were extracted from included publications. The data comprised the name of author, date of publications, mean age of case and control group, the study type of comparison/contrast, and allelic and genotype frequencies. The flow diagram of literature review and study selection is shown in figure 1.

As previous investigations on SNPs of TGF-β at codon 10 and 25 were scant, further analyses on these studies (3 studies, two on codon 10 and one on codon 25) were not done.

#### Quality assessment and risk of bias:

Quality assessment was performed using the Newcastle-Ottawa Scale (NOS), for observational case-control studies 32. The NOS assesses three main aspects of observational studies including sample selection, comparability of cases and controls, and exposure. Using this scale, studies with scores less than 4 stars have the highest risk of bias and the lowest quality. After independent review of all included studies by two reviewers, none of them scores below 4 in NOS scale.

#### Meta-analysis:

Meta-analysis was performed using Review Manager (version 5.3. Copenhagen: The Nordic Cochrane Centre, the Cochrane Collaboration, 2014), on the above selected five studies 33–37, and data from the current study (Table 1). For individual studies, allelic or genotypic frequencies were extracted and OR (95%CI) was reported. For the whole group, pooled OR of the genotypes using random- or fixed-effects models was calculated. Fixed effects and random effects were interchangeably used as the analysis model. Heterogeneity was determined using Q statistic tests and the *I^2^* index. According to the Cochrane guidelines, the *I^2^* less than 40% means that the inconsistency across studies is not important. In this case, fixed effects model was used. If the *I^2^* estimates fluctuated more than 40%, the random effects procedure was used as the analysis model. A p-value less than 0.05 was considered statistically significant.

**Table 1.** Studies included in meta-analysis for IL-10 single nucleotide polymorphisms in Kawasaki Disease (KD)

**Articles addressing IL-10 polymorphisms with KD risk**									
**First Author**	**Year**	**No. of KD**	**No. of HC**	**Age in PD**	**Age in HC**	**M/F in PD**	**M/F in HC**	**Study population**	**Comparing**
**Hsueh**	2009	105	277	3.1±2	-	61/44	-	Taiwan	KD *vs*. HC
**Weng**	2010 Nov	211	321	2.2±2	48.9±12.2	130/81	134/87	Taiwan	KD *vs*. HC
**Weng**	2010 May	140 (33 chronic, 107 acute)	418	2.52±2.84	2.47±2.49	96/44	242/176	Taiwan	KD+CAA *vs*. KD-CAA
**Hsieh**	2011	146	100	2.37±2.46	14.5±0.5	64/82	181/134 Taiwan		KD *vs*. HC
**Lin**	2014	58	315	1.25	-	41/17	-	Taiwan	KD with CAA *vs*. HC
**Our study**	2018	110	140	3.29±2.36	3.04±2	60/55	70/70	Iran	KD *vs*. HC

* KD: Kawasaki Disease; CAA: Coronary Artery Aneurysm; HC: Healthy Controls; M/F: Male to Female Ratio.

## Results

### IL-10 polymorphisms at −1082, −819, or −592 are not associated with KD risk in Iranian population

In the first part of the study, a matched-case-control research on Iranian patients with KD (mean age: 3.29±2.36 years; 60 male/55 female) and a group of age-sex matched healthy controls (mean age: 3.04±2 years; 70 males/70 females) was done. Allele frequencies of SNPs of TGF-β, IL-10 in the two groups are presented in table 2, which shows no significant association between patients and controls group. Similarly, none of the genotypic variants of TGF-β or IL-10 reached significance level in association with KD in Iranian population (Table 2). The only significant difference in haplotype variants of IL-10 and TGF-β was haplotype TC at codons 10 and 25 which was over-represented in patients compared to controls (Table 3).

**Table 2.** Allele and genotype frequencies of TGF-β and IL-10 in patients with KD and controls

**Cytokine**	**Position**	**Alleles**	**Kawasaki (n^†^110)**	**Control (N=140)**	**p-value**	**OR (95%CI)**
**TGF-B**	Codon 10	CC	16(14.5%)	21(15%)	0.96	**0.88(0.31–2.38)**
		CT	76(69.1%)	91(65%)	0.67	**1.23(0.59–2.57)**
		TT	18(16.4%)	28(20%)	0.79	**0.82(0.33–2.01)**
		C	108(49.1%)	133(47.5%)	0.99	**1.03(0.64–1.64)**
		T	112(50.9%)	147(52.5%)	0.99	**0.97(0.61–1.56)**

**TGF-B**	Codon 25	CC	4(3.6%)	3(2.2%) 1.00		**1.31**
		GC	20(18.2%)	17(12.1%)	0.35	**1.66(0.65–4.19)**
		GG	86(78.2%)	120(85.7%)	0.33	**0.61(0.25–1.50)**
		C	32(14.5%)	23(8.2%)	0.34	**1.55(0.69–3.46)**
		G	188(85.5%)	257(91.8%)	0.34	**0.65(0.29–1.46)**

**IL-10**	−1082	AA	28(25.5%)	53(37.8%)	0.16	**0.57(0.27–1.21)**
		GA	72(65.5%)	75(53.6%)	0.13	**1.73(0.86–3.52)**
		GG	10(9.1%)	12(8.6%)	1.0	**0.85(0.22–3.04)**
		A	128(58.2%)	181(64.6%)	0.38	**0.80(0.49–1.29)**
		G	92(41.8%)	99(35.4%)	0.38	**1.26(0.78–2.03)**

**IL-10**	−819	TT	28(25.5%)	53(37.8%)	0.16	**0.57(0.27–1.21)**
		CT	72(65.5%)	75(53.6%)	0.13	**1.73(0.86–3.52)**
		CC	10(9.1%)	12(8.6%)	1.0	**0.85(0.22–3.04)**
		C	148(67.3%)	199(71.1%)	0.71	**0.89(0.53–1.48)**
		T	72(32.8%)	81(28.9%)	0.71	**1.13(0.68–1.88)**

**IL-10**	−592	AA	8(7.3%)	9(6.4%)	1.0	**1.07(0.31–3.49)**
		CA	56(50.9%)	63(45%)	0.17	**1.62(0.83–3.09)**
		CC	46(41.8%)	68(48.6%)	0.15	**0.60(0.30–1.19)**
		A	72(32.8%)	81(28.9%)	0.25	**1.35(0.82–2.21)**
		C	108(67.3%)	199(71.1%)	0.25	**0.74(0.45–1.22)**

† n, denotes number of participants in each group. Bold values signify p<0.05.

OR: odds ratio, CI; confidence interval.

**Table 3.** Haplotype frequencies of TGF-β and IL-10 in patients with KD and controls

**Cytokine**	**Haplotype**	**Kawasaki (n^†^=110 )**	**Control (n=140)**	**p-value**	**OR (95%CI)**
**TGF-β (codon10, codon25)**					
	CG	90(40.9%)	110(39.3%)	0.85	1.07(0.66–1.73)
	TG	102(46.4%)	147(52.5%)	0.40	0.81(0.5–1.29)
	CC	18(8.2%)	23(8.2%)	0.21	0.52(0.19–1.36)
	TC	10(4.5%)	0(0%)	0.00	-
**IL-10 (**−**1082,** −**819,** −**592 )**					
	GCC	92(41.8%)	99(35.3%)	0.35	1.28(0.78–2.09)
	ACC	56(25.5%)	100(35.7%)	0.11	0.65(0.38–1.10)
	ATA	72(32.8%)	81(28.9%)	0.60	1.17(0.70–1.97)

† n, denotes number of participants in each group. Bold values signify p<0.05.

### IL-10 polymorphisms at −1082, −819, or −592 are not associated with KD or KD with CAA by meta-analysis

Review of literature yielded five studies, investigating association of IL-10 polymorphisms in KD patient. Total number of patients included from previous studies were 660 patients *vs.* 1331 Healthy Controls (HC), added to 110 patients and 140 controls from our study, yielding a total number of 770 KD patients and 1471 HC for the meta-analytic population. Mean age of patients ranged from 1.25 to 3.29±2.36 years and Taiwan studies were divided into those comparing frequency of polymorphisms in KD patients *vs.* healthy controls, including results from current study, and those comparing polymorphisms in KD patients with CAA and those without CAA. One study had only addressed polymorphic variants at −592 (C/T) (33). Also, Weng *et al* reported results of their study in Thai population in two separate publications 35,36, with different exposures and outcomes (KD *vs.* HC and KD with CAA *vs.* KD without CAA). Total number of subjects in pooled analysis for KD *vs.* HC comparison was 572 patients and 776 HC, and 272 *vs*. 726 for KD+ CAA *vs*. KD-CAA contrast.

Results of meta-analysis based on odds ratio revealed no significant difference in polymorphic variants of IL-10 at −1082, −819, or −592, in either allelic or genotypic variants, comparing KD *vs.* healthy individuals. The same result was attributed to subgroup meta-analysis of KD+CAA *vs.* KD without CAA (Supplementary figures of 1–3). According to the preliminary negative results, no further meta-regression analysis using existing covariates including age and sex of the subjects was performed.

**Figure:**
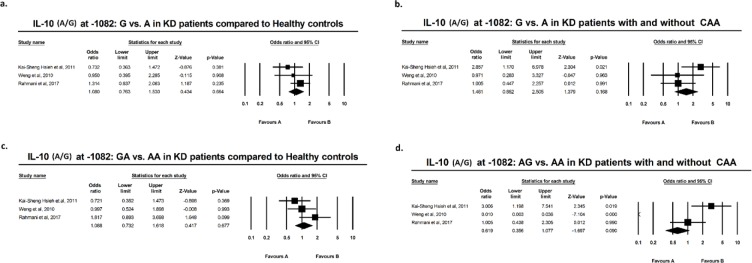
Suppl 1

**Figure:**
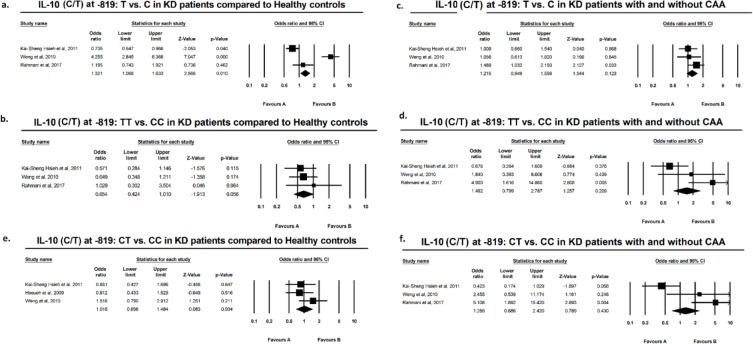
Suppl 2

**Figure:**
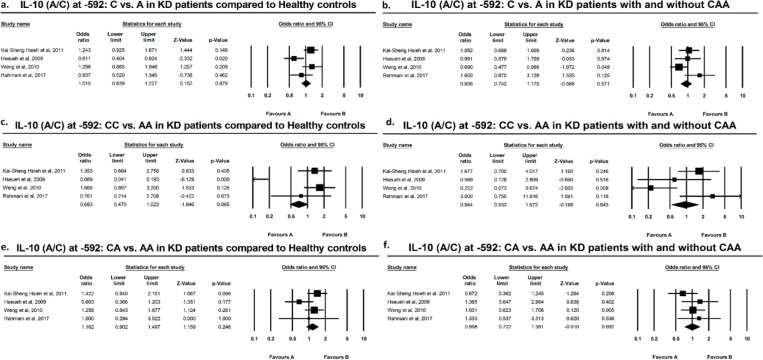
Suppl 3

## Discussion

Results of the current study show that despite individual reports suggesting an association between IL-10 cytokines, none of the polymorphic variants at −1082, −819, or −592 of the gene predicts risk of KD or CAA formation. Cytokine levels in acute phase of vasculitides, either in serum or as cytokine expression in peripheral blood, reflect previous or current status of immune network. Expression of cytokines in various phases of vasculitis predicts direction of immune cell maturation, T-cell subset polarization, and persistence of inflammation *vs.* initiation of repair and remodelling phenomena.

There is no doubt that genetic and ethnic susceptibility is a major contributing factor to KD pathology, with siblings of KD patients being at a 10–30 fold higher risk for developing KD and Japan, Korea and Taiwan reporting the highest annual incidence rates 38. On the contrary, clustering of new cases during spring and summer and evidence of aberrant immune function in response to infection support an infectious pathology to this disorder.

Acute stage of KD is usually symptomatic, with mucocutaneous manifestations, release of vascular endothelial antigens and pro-inflammatory cytokine release, and finally abrupt onset of high spiking fever 3. Peripheral expression of acute phase cytokines such as IL-6, TNF-α and IFN-γ as well as IL-17 and interestingly IL-10 are elevated in acute phase KD. T-cell subset analysis displays a picture of Th17 over Treg expansion and predominance of CD4+ subset over CD8+ in acute KD.

Fortunately, KD is self-limiting in nature, which resolves along with improvement of clinical picture. Meanwhile, transition to chronic stage KD might happen among up to 30% of untreated patients 38. Resolution of acute systemic response is mirrored in Th17 decrease in proportion and Treg expansion is reflected in FOXP3 upregulation. Acute phase KD is therefore associated with Th17 over Treg imbalance 38, and the convalescent period is characterized by a reversal in this ratio. Successful response to IVIG resolution is similarly associated with Th17 cytokines, IL-17 and IL-23, downregulation and an increase in Treg subset, which is not mirrored by concomitant IL-10 and TGF-β expression 8. Resistance to IVIG therapy is therefore anticipated by a failure of Treg expansion and persistent high levels of IL-10 and IL-17 expression.

Chronicity and IVIG resistance in KD patients confer greater risks for coronary artery aneurysm formation. Myocardial infarction is the leading cause of mortality in KD, resulting from aneurysm rupture or premature atherosclerosis 3. Research focuses have turned eyes onto early identification of markers of KD resistance to intravenous immunoglobulin infusion and aneurysm formation. IL-10 and IL17 are the two mostly investigated cytokines regarding CAA risk in KD. With suppression of Treg cell line in acute phase KD, there might exist an unusual source of secretion for high IL-10 and TGF-β levels. In acute phase KD 9, TGF-β together with IL-10 and/or IL-21, are known to drive Th17 line maturation by activation of specific ROR-γt transcription factor 8. Increased levels of these two cytokines might thus reflect Th17 expansion and further demonstrate a distinct active role for IL-17 in acute KD pathogenesis. High levels of IL-10 have been linked to abdominal aortic aneurysm formation, where the IL-10 genotype AA at −1082 increases risk of aneurysm formation 39. A balance between pro-inflammatory and anti-inflammatory cytokine networks is further regarded to regulate aneurysm stability or rupture. With high levels of IL-10 being a feature of acute phase KD, a number of studies have addressed whether IL-10 polymorphic variants are able to predict risk for CAA in KD 33–35.

While Hsueh *et al* reported no significant association at rs1800872 (−592, A/C), Wang *et al* reported higher frequency of C allele and CC gene in KD patients with coronary artery lesions in acute but not chronic stage KD 36. Lin *et al* 34 found higher frequency of G allele and GG genotype at −1082 in KD with CAA and Wang reported the same results with C/CC at −819. Meta-analysis by direct pooling of subjects from these studies, all from Thai population, yielded no significant results (Supplementary figures 1–3). Pooling data from the current study and previous studies demonstrated that mentioned IL-10 polymorphisms do not confer predilection nor do they confer protection, with regard to KD or KD with CAA.

TGF-β is able to induce vascular wall myofibroblast generation 27, alter matrix metalloproteinase-9 activity, and result in collagen lattice fragmentation and loss of medial layer elastin, providing an underlying pathology for CAA formation 40.

Although literature on TGF-β polymorphisms in KD patients is scant, polymorphisms in components of the TGF-β/SMAD signalling pathway greatly influence susceptibility to KD 13–16, TGF-β signalling pathway components like TGFBR1 and TGFBR2 are directly involved in aortic aneurysm progression and coronary artery aneurysm formation 41. No significant association was found between TGF-β polymorphisms at rs1982073 and rs1800471 with KD susceptibility.

## Conclusion

Common SNPs of TGF-β and IL-10 in patients with KD were investigated in our sample of Iranian population, which revealed no significant results in any of the allelic, genotypic or haplotypic components. Previous studies had reported conflicting results on association of IL-10 polymorphisms at −1082, −819, or −592 and TGF-β polymorphisms at codon 10 and codon 25, with KD and/or coronary artery aneurysm as its most lethal complication. Through an updated meta-analysis, no association was found between the mentioned SNPs and risk of KD or CAA. Although previous reports have suggested a dysregulation in anti-inflammatory cytokines as a mechanism for CAA formation, but these results are not supported by our meta-analysis results on common polymorphisms of TGF-β and IL-10. One major limitation of the study to be addressed is that studies that met our inclusion criteria had all taken place in Taiwan and this might limit extrapolation of the results.
